# Color stability of ceramic veneers as a function of resin cement curing mode and shade: 3-year follow-up

**DOI:** 10.1371/journal.pone.0219183

**Published:** 2019-07-01

**Authors:** Janes Francio Pissaia, Brunna Katyuscia de Almeida Guanaes, Cibele Cândida de Almeida Kintopp, Gisele Maria Correr, Leonardo Fernandes da Cunha, Carla Castiglia Gonzaga

**Affiliations:** School of Health Sciences, Graduate Program in Dentistry, Universidade Positivo, Curitiba, PR, Brazil; University of Montreal, CANADA

## Abstract

The influence of curing mode and shade of resin cements on the color stability of minimum-thickness ceramic veneers after a three-year storage time in distilled water was evaluated in this study. Ninety-six 0.5-mm-thick feldspathic ceramic veneers (Mark II) were luted onto resin composite substrates (Filtek Z350 XT, shade A2E) with two light-cured (NX3 Light-cure and AllCem Veneer) and dual-cured resin cements (NX3 dual-cure and AllCem) in various shades. The specimens were stored in distilled water at 37°C. Color measurements were performed with a spectrophotometer at the following times: 1h and 24h; 7, 30, and 180 days; and 1, 2, and 3 years. Data for color difference (ΔE_ab_) light-cured and dual-cured resin cements were analyzed by two-way ANOVA with repeated measures and Tukey’s test (α = 0.05). For the light-cured cements, the ΔE_ab_ values were as follows: NX3-Yellow (2.37±1.35) = ACV-A1 (2.40±1.21) = ACV-Trans (2.52±1.46) = ACV-E-Bleach M (2.56±1.42) = NX3-White (2.69±1.49) = NX3-Clear (2.98±1.68). The lowest ΔE values were found for 1 h (0.61±0.36)^a^, followed by 24 h (1.15±0.55)^b^ and 30 days (2.48±1.11)^c^. One year, 180 days, and 2 and 3 years presented higher ΔE_ab_ values (3.34±0.94, 3.52±1.04, 3.52±0.95 and 3.55±1.14, respectively)^d^. For the dual-cured cements, the ΔE_ab_ values varied as follows: NX3-Clear (2.32±1.24)^a^ = NX3-Yellow (2.37±1.32)^a^ = NX3-White (2.76±1.43)^a^ < AC-Trans (3.77±1.91)^b^ = AC-A3 (4.13±2.11)^b^ < AC-A1 (5.38±2.92)^c^. Considering time, the lowest ΔE_ab_ values were found for 1 h (0.48±0.25)^a^, followed by 24 h (2.13 ±0.83)^b^, 30 days (3.54±1.31)^c^, and 180 days (3,70±1.73)^c^. The follow-up times of 1 (4.43±2.15)^d^, 2 (5.02±1.74)^e^, and 3 years (4.90±2.12)^e^ presented higher ΔE_ab_ values. This study demonstrated that light-cured resin cements were less susceptible to color change than dual-cured cements. After 2 years of follow-up, all cements presented ΔE_ab_ values above the acceptability threshold.

## Introduction

Ceramic laminate veneers are among the most desired cosmetic treatments because they present the possibility of lighter teeth, preparation with minimal wear, the ability to change shape, and improved aesthetics [1]. Currently, there is a wide variety of dental ceramics available for indirect restorations that can be used for the manufacture of thin laminate veneers, including conventional sintered or machined feldspathic ceramics, heat-pressed or machined glass-ceramics, and some resin-matrix ceramics, such as resin nanoceramics (e.g., Lava Ultimate) and glass-ceramic in a resin interpenetrating matrix (e.g., Vita Enamic) [2, 3].

All-ceramic restorations with a high degree of translucency, such as feldspathic ceramics and glass-ceramics, allow the passage and scatter of light, especially at small thicknesses [4]. In these cases, there is a great influence of the dental substrate color on which they are cemented, as well as the material used for cementation [5, 6]. The luting material plays a major role in the aesthetic outcome of ceramic veneers, allowing good shade matching with adjacent teeth [7]. Thus, changes in the color of resin cement used for luting may become visible, affecting the final aesthetic appearance of the restoration and leading to treatment failure [8].

For the cementation of all-ceramic restorations, resin-based cements are generally used because they can be adhesively bonded to dental structures and they exhibit low solubility, good mechanical properties, and favorable aesthetics [9]. Resin cements are usually divided into three categories, according to their curing mode: chemically activated, light-cured, and dual-cured cements. Chemically activated cements are mostly employed for cementation of metallic and metal-ceramic restorations or cast posts. Light-cured cements have a more restricted indication, used only for the luting of laminate veneers because of the decrease in light intensity during transmission through the restoration [10]. Dual-cured cements were developed to obtain good mechanical properties and a high degree of conversion in either the presence or absence of light [11, 12]. However, regarding the color stability of resin cements, for chemically activated and dual-cured materials, the oxidation of the reactive groups present in the tertiary amines may cause a color change in the cement over time [5].

The final color of thin ceramic restorations is determined by a combination of the substrate, the thickness of the ceramic, and the luting material [13, 14]. Among these factors, resin cement is the one that can have the most influence on the final color of ceramic laminate veneers [4]. However, depending on the curing mode and commercial brand, cements identified with the same shade (A1, A2, translucent, bleach, etc.) do not have the same color parameters [14]. Therefore, these variations can influence the color stability of the cement and the final color of ceramic restorations [15].

It is important to note that the influence of different shades of dual-cured and light-cured cements underlying ceramic restorations and their long-term discoloration is little known. Also, this discoloration becomes much more important beneath thin-translucent ceramic veneers [5]. Because there are few studies on the long-term (more than one year) color stability of cemented thin ceramic veneers with resin cements having various shades and curing modes [16], the objective of this study was to evaluate the color stability of cemented thin ceramic laminate veneers as a function of the curing mode and shade of resin cements during a three-year follow-up period. The null hypotheses were as follows: i) the curing modes and the different shades of resin cements would not influence the color stability of the cemented thin ceramic veneers; and ii) long-term storage in distilled water would not influence the color stability of the cemented thin ceramic veneers.

## Materials and methods

Ninety-six disk specimens with 6 mm in diameter and 1 mm in height were prepared with composite resin (shade A2E, Filtek Z350 XT, 3M, St. Paul, MN, USA). Each specimen was prepared by inserting the composite resin in a Teflon mold in a single layer. All specimens were light-cured with an LED curing unit (Dental Woodpecker LED, China), with irradiance of 1200 mW/cm^2^, for 20 s. The irradiance of the light source was previously measured by a radiometer (LED Demetron, Kerr, Middleton, WI, USA). After photo-activation, the specimens were carefully removed from the matrix. These composite resin disks served as substrates for cementing the thin ceramic veneers.

Five blocks of feldspathic ceramic (shade 2M1, VITABLOCS Mark II, Vita Zahnfabrik, Bad Säckingen, Germany) were sliced into 0.5-mm-thick veneers using a low-speed diamond saw (IsoMet 1000, Buehler, Lake Bluff, IL, USA) under refrigeration.

For cementation on the composite resin substrate, the ceramic veneers were conditioned with 10% hydrofluoric acid (Condac Porcelana, FGM, Joinville, Brazil) for 60 s, washed with running water for 20 s, and dried. A layer of silane coupling agent (Prosil, FGM, Joinville, SC, Brazil) was applied for 60 s, followed by a layer of hydrophobic adhesive (Scotchbond MultiPurpose, 3M, St. Paul, MN, USA).

The composite resin disks were cleaned with 37% phosphoric acid (Condac 37, FGM, Joinville, Brazil) for 20 s, washed for 20 s in running water, and dried. A layer of hydrophobic adhesive (Scotchbond Multipurpose, 3M, St. Paul, MN, USA) was applied and light-cured for 10 s. The ceramic veneers were then cemented onto the composite resin discs with two light-cured resin cements or two dual-cured cements with various shades (Table 1) (n = 8). The cementation was carried out by digital pressure to simulate clinical conditions. Each specimen was photo-activated for 20 s. The specimens were stored in distilled water at 37°C throughout the period of color stability evaluation.

**Table 1:** Curing mode, shades, and composition of the resin cements.

Resin cement	Curing mode	Composition	Shade
NX3 Light-cure (Kerr, Orange, CA, USA)	Light-cured	Uncured methacrylate ester monomers, inert mineral fillers, free tertiary amines, benzoyl peroxide, stabilizers, radiopaque agent, glycerin, water, fumed silica, and inert glass powder	Clear
			White
			Yellow
NX3 Dual-cure (Kerr, Orange, CA, USA)	Dual-cured	Uncured methacrylate ester monomers, HEMA, PTU, CHPO, free tertiary amines and benzoyl peroxide, inert mineral fillers, titanium dioxide, radiopaque agent, and pigments	Clear
			White
			Yellow
Allcem Veneer–ACV (FGM, Joinville, SC, Brazil)	Light-cured	Methacrylate monomers, camphorquinone, co-initiators, stabilizers, pigments, silanized barium-alumino-silicate glass particles, and silicon dioxide.	Trans
			E-Bleach M
			A1
Allcem–AC (FGM, Joinville, SC, Brazil)	Dual-cured	Bis-GMA, Bis-EMA, TEGDMA, co-initiators, initiators (camphorquinone and dibenzoyl peroxide), stabilizers, barium-alumino-silicate glass particles, and silicon dioxide	Trans
			A1
			A3

HEMA: 2-hydroxyethyl methacrylate; PTU: pyridyl thiourea; CHPO: cumene hydroperoxide; Bis-GMA: bisphenol A diglycidyl ether dimethacrylate; Bis-EMA: bisphenol A ethoxylated dimethacrylate; TEGDMA: triethylene glycol dimethacrylate.

The color measurements were performed with a spectrophotometer (EasyShade Advance, Vita Zahnfabrik, Bad Säckingen, Germany) according to the CIELab (Commision Internationale de l’Eclairage, L*, a*, b*) coordinates. The CIE L* parameter corresponds to the degree of lightness and darkness, whereas a* and b* coordinates correspond to the amount of red or green (+a* = red, -a* = green) and yellow or blue (+b* = yellow, -b* = blue), respectively. Baseline color parameters were determined 10 min after photo-activation and over a white background. The CIELab coordinates were used to calculate the color difference (ΔE_ab_) between the “before” and “after” periods of 1 h, 24 h, 7 days, 30 days, 180 days, 1 year, 2 years, and 3 years. The color difference (ΔE_ab_) between the baseline and subsequent readings was calculated according to the equation [17]:

$$\Delta E_{ab}=[{\left(\Delta L*\right)}^{2}+{\left(\Delta a*\right)}^{2}+{\left(\Delta b*\right)}^{2}{]}^{1/2}$$

where ΔL*, Δa*, and Δb* are the differences between the parameters before (*baseline*) and after each period of time. The 50:50% perceptibility threshold and the 50:50% acceptability threshold for CIELab (ΔE_ab_) are 1.22 and 2.66, respectively [18].

The ΔE_ab_ results for light-cured and dual-cured resin cements were analyzed by two-way ANOVA with repeated measures and Tukey’s HSD test (α = 0.05).

## Results

The mean values of ΔE_ab_ with standard deviations are presented in Tables 2 and 3 for light-cured and dual-cured resin cements.

**Table 2:** Means and standard deviations for ΔE_ab_ values of the light-cured resin cements.

Resin cement	Shade	ΔE_ab_						
		1 h	24 h	30 days	180 days	1 year	2 years	3 years
NX3 Light-cure	Clear	0.46 ± 0.19 aA	1.29 ± 0.28 aA	3.43 ± 1.23 aB	4.61 ± 1.14 bB	3.62 ± 0.78 aB	3.66 ± 0.97 aB	3.78 ± 1.33 aB
	White	0.80 ± 0.24 aA	0.92 ± 0.27 aA	3.04 ± 0.87 aB	3.13 ± 0.79 abB	2.83 ± 0.92 aB	4.03 ± 0.95 aB	4.09 ± 1.21 aB
	Yellow	0.49 ± 0.29 aA	0.73 ± 0.58 aA	2.11 ± 0.27 aAB	2.48 ± 0.35 aB	2.83 ± 0.34 aB	4.24 ± 0.45 aC	3.73 ± 0.93 aBC
AllCem Veneer (ACV)	Trans	0.57 ± 0.26 aA	1.45 ± 0.39 aAB	2.16 ± 1.41 aABC	3.32 ± 0.81 abCD	3.92 ± 1.39 aD	3.38 ± .73 aCD	2.85 ± 1.05 aBCD
	E-Bleach M	0.65 ± 0.48 aA	1.02 ± 0.45 aA	1.87± 0.30 aAB	3.96 ± 0.53 abC	3.76 ± 0.50 aC	2.82 ± 0.95 aBC	3.77 ± 0.91 aC
	A1	0.69 ± 0.47 aA	1.46 ± 0.80 aAB	2.17 ± 1.02 aABC	3.49 ± 0.97 abC	2.97 ± 1.49 aBC	3.05 ± 0.54 aBC	3.01 ± 0.70 aBC

In each row, values followed by the same capital letters are statistically similar (p > 0.05). In each column, values followed by the same lowercase letters are statistically similar (p > 0.05).

**Table 3:** Means and standard deviations for ΔE_ab_ values of the dual-cured resin cements.

Resin cement	Shade	ΔE_ab_						
		1 h	24 h	30 days	180 days	1 year	2 years	3 years
NX3 Dual-cure	Clear	0.31 ± 0.12 aA	1.33 ± 0.32 aAB	2.78 ± 0.53 aBC	2.52 ± 0.85 aBC	2.40 ± 0.76 aBC	3.65 ± 0.74 aC	3.25 ± 0.74 aC
	White	0.52 ± 0.13 aA	1.47 ± 0.57 aAB	3.33 ± 1.41 abCD	2.56 ± 0.29 aBC	3.44 ± 0.41 aCD	4.50 ± 0.66 abD	3.51 ± 0.53 aCD
	Yellow	0.37 ± 0.23 aA	1.89 ± 0.39 aAB	2.33 ± 0.25 aBC	2.08 ± 0.67 aAB	2.02 ± 0.48 aAB	3.89 ± 0.95 abC	4.00 ± 1.02 abC
AllCem	Trans	0.56 ± 0.20 aA	2.35 ± 0.60 abAB	4.73 ± 1.66 bCD	3.80 ± 1.01 abBC	5.60 ± 0.45 bC	4.86 ± 1.17 abCD	4.52 ± 1.19 abCD
	A1	0.36 ± 0.11 aA	3.38 ± 0.48 bB	4.56 ± 0.42 bBC	5.98 ± 0.79 cC	7.29 ± 1.33 bCD	7.71 ± 1.47 cD	8.42 ± 1.75 cD
	A3	0.74 ± 0.30 aA	2.36 ± 0.52 abAB	3.52 ± 0.58 abBC	5.22 ± 1.53 bcCD	5.83 ± 1.30 bD	5.53 ± 1.21 bD	5.70 ± 1.24 bD

In each row, values followed by the same capital letters are statistically similar (p > 0.05). In each column, values followed by the same lowercase letters are statistically similar (p > 0.05).

For the light-cured cements, the statistical analysis showed no significant differences for resin cement (p = 0.3130). Time and the double interaction were statistically significant (p < 0.0001). For the resin cement, the ΔE_ab_ values were as follows: NX3 Yellow (2.37 ± 1.35) = ACV A1 (2.40 ± 1.21) = ACV Trans (2.52 ± 1.46) = ACV E-Bleach M (2.56 ± 1.42) = NX3 White (2.69 ± 1.49) = NX3 Clear (2.98 ± 1.68). For time, the lowest ΔE_ab_ values were found for 1 h (0.61 ± 0.36)^a^, followed by 24 h (1.15 ± 0.55)^b^ and 30 days (2.48 ± 1.11)^c^. The time periods of 1 year, 180 days, 2 years, and 3 years presented higher ΔE_ab_ values (3.34 ± 0.94, 3.52 ± 1.04, 3.52 ± 0.95 and 3.55 ± 1.14, respectively)^d^.

For the dual-cured resin cements, the statistical analysis showed significant differences for resin cement, time, and the double interaction (p < 0.0001). Regarding the cement, NX3 presented lower ΔE_ab_ values than did AC, and the ΔE_ab_ values varied as follows: NX3 Clear (2.32 ± 1.24)^a^ = NX3 Yellow (2.37 ± 1.32)^a^ = NX3 White (2.76 ± 1.43)^a^ < AC Trans (3.77 ± 1.91)^b^ = AC A3 (4.13 ± 2.11)^b^ < AC A1 (5.38 ± 2.92)^c^. Considering time, the lowest ΔE_ab_ values were found for 1 h (0.48 ± 0.25)^a^, followed by 24 h (2.13 ± 0.83)^b^, 30 days (3.54 ± 1.31)^c^, and 180 days (3,70 ± 1.73)^c^. The follow-up times of 1 year (4.43 ± 2.15)^d^, 2 years (5.02 ± 1.74)^e^, and 3 years (4.90 ± 2.12)^e^ presented higher ΔE values.

Data analysis showed that ΔE_ab_ changes above the 50:50% acceptability threshold (2.66 units) started after 24 h of follow-up for AllCem A1 (dual-cured); after 30 days for NX3 Clear and NX3 White (light-cured and dual-cured), and AllCem Trans and A3 (dual-cured); after 180 days for all shades of AllCem Veneer (light-cured); and after 1 year days for NX3 Yellow (light-cured). After 2 years of follow-up, all cements presented values of ΔE_ab_ above 50:50% acceptability threshold.

## Discussion

The color stability of resin cements is important because the short- or long-term color change may render the result of the aesthetic treatment unfavorable. All the null hypotheses tested in this study were rejected because curing mode, shade of resin cement, and storage time influenced the color stability of thin ceramic laminates after cementation.

Turgut *et al*. [5] suggested that resin cement is the main cause of color change in restorative treatments with ceramic laminate veneers, as ceramic materials have high color stability. Color changes with ΔE_ab_ values above 2.66 are considered clinically perceptible [18], which may compromise the treatment aesthetical outcome. The change in color stability of the cement appears to be related to its chemical composition, type of initiators and inhibitors of the polymerization reactions, degradation of residual amines, and the oxidation of carbon double bonds that were not converted into polymers [9].

In the present study, when comparing the different resin cements, it is important to note that the light-cured cements remained for longer periods of time below the 50:50% acceptability threshold of 2.66 when compared to the dual-cured cements. For light-cured materials, AllCem Veneer remained at acceptable ΔE_ab_ values for up to 180 days. For the NX3 light-cured, after 30 days, two of the three groups (those in lighter shades, Clear and White), had already exceeded the 50:50% acceptability threshold. For the dual-cured cements, after 30 days of follow-up, five of the six groups (except for NX3 Yellow) presented ΔE_ab_ values above the 2.66 threshold. After 2 years, all cements had a ΔE_ab_ value higher than 2.66 units. The difference in color stability between the cements may be associated with the chemical composition of the materials. There are no major differences in the chemical compositions of AllCem and AllCem Veneer, which could explain the similar performance with respect to color stability. NX3 Light-cure and NX3 dual-cure have different components in their composition, and this factor may be related to their improved color stability in relation to Allcem and AllCem Veneer. According to the manufacturer's information, AllCem cement has methacrylate monomers such as TEGDMA and Bis-GMA in its basic composition. When these two monomers are combined in aqueous media, the sorption of water is directly proportional to the concentration of TEGDMA, which contributes to the release of monomers and color change of the cement [19, 20].

The higher ΔE_ab_ values for the dual-cured cements can be attributed to the presence of tertiary aromatic amines and benzoyl peroxide as an initiator system. The degradation of the residual amines and the oxidation of unreacted carbon double bonds in the polymerization reaction tend to darken the cements over time. The light-cured cements have aliphatic amines in their chemical composition, which makes them less susceptible to color change [21]. The presence of camphorquinone in the composition of the light-cured cements is responsible for the initiation of the polymerization reaction; however, when this yellowish compound is not completely consumed, it is degraded and causes a color change in the cement over time [22].

Ural *et al*. [23] have shown that the inclusion of an initiation system with free tertiary amines and benzoyl peroxide, such as NX3, makes a cement more prone to color stability. In their study, third-generation adhesive resin cements with free tertiary amines and benzoyl peroxide underwent smaller color changes than the light- and dual-cured resin cements tested. The present study is also in agreement with the results of Smith *et al*. [16], who evaluated the difference in color stability of resin cements in three activation modes after one year of storage in water and observed that NX3 cement had the lowest ΔE_ab_ values, regardless of the activation mode. In the present study, for dual-cured cements, NX3 presented higher color stability than AllCem. This result can be attributed to the fact that NX3 contains an amine-free redox initiator system and an optimized resin matrix.

The shade is also a factor that influences the color stability of resin cements. Previous studies have shown that lighter shades tend to have lower color stability than darker shades, producing more visible changes and higher ΔE_ab_ values [15, 24]. The results of this study showed that the two lighter shades (clear and white) of the NX3 light-cured and dual-cured resin cements presented higher ΔE_ab_ values (above the 50:50% acceptability threshold of 2.66) after 30 days of follow-up. In contrast, the darker shade (yellow) of these cements, showed ΔE_ab_ values above the acceptability threshold after 1 or 2 years of follow-up. The color change of the cements with various shades could be related to the differences in the amount of opacifiers and inorganic fillers present in the composition, producing different refractive indices [25]. A recent systematic review of in vitro studies evaluated the influence of light-cured luting materials and associated factors on the color of ceramic veneers, and concluded that, regarding the shade of luting resin cements, translucency and value showed the greatest visible color differences for ceramic veneers. Also, the effect of resin cement shade on the final color of veneers was significantly affected by the thickness and opacity of the ceramic used for the restoration [7].

The aging of the specimens, carried out in distilled water at a constant temperature of 37°C, may have contributed to the color change [20], as the resin cements are prone to the hydrolysis of polymer bonds caused by water absorption, leading to the hydrolytic degradation of the polymer matrix [26, 27].

Few clinical studies have evaluated the color changes and marginal discoloration of thin ceramic laminate veneers cemented with light- and dual-cured resin cements. Marchionatti *et al*. [28], in a split-mouth randomized clinical trial with 0.3-mm-thick ceramic laminate veneers, reported that the color stability of ceramic laminate veneers was similar for both curing modes for all evaluated periods. Also, for both materials, marginal discoloration increased over a 2-year period.

The increased demand for aesthetic treatments with ceramic laminates requires careful consideration by clinicians when choosing the luting material, as it is extremely important to the success of the treatment over the long term. To date, few studies have evaluated the color stability of thin ceramic laminates after cementation after long-term follow-up. Despite the limitations of this in vitro study, the results can help clinicians in their choice of resin cement when luting low-thickness ceramic laminates.

## Conclusions

It can be concluded that resin cements, activation mode, and time influenced the color stability of cemented thin ceramic laminate veneers. For dual-cured cements, NX3 cement showed better color stability than did AllCem. Light-cured resin cements were less susceptible to staining and color change than dual-cured cements. After 2 years of follow-up, all cements presented values of ΔE above the 50:50% acceptability threshold.

## Supporting information

S1 File Results (raw data) for ΔE_ab_ values of the light-cured and dual-cured resin cements. (PDF)

S2 File Statistical results for the light-cured resin cements. (PDF)

S3 File Statistical results for the dual-cured resin cements. (PDF)

S4 File Certificate of English editing. (PDF)
