# Supplementary material for: Color stability of ceramic veneers as a function of resin cement curing mode and shade: 3-year follow-up
Source: PLoS One. 2019 Jul 1;14(7):e0219183. doi: 10.1371/journal.pone.0219183 (PMC6602287; doi:10.1371/journal.pone.0219183)
Supplement: S1 File — (PDF) [file pone.0219183.s001.pdf]

Color stability of ceramic veneers as a function of resin cement curing mode and shade: 3-year follow-up

		ΔE_{ab} 1 h	ΔE_{ab} 24 h	ΔE_{ab} 7 days	ΔE_{ab} 30 days	ΔE_{ab} 180 days	ΔE_{ab} 1 year	ΔE_{ab} 2 years	ΔE_{ab} 3 years
NX3 Light-cure White									
1		0.57	0.24	0.89	1.14	1.61	2.85	2.77	3.25
2		1.00	1.18	2.03	3.64	3.44	1.39	2.62	2.04
3		0.42	0.81	1.89	3.81	3.45	4.18	4.79	4.03
4		1.16	1.07	2.75	3.80	3.73	3.26	4.19	4.43
5		0.99	1.00	1.41	3.03	2.25	1.70	3.77	2.83
6		0.62	1.04	2.53	3.72	3.65	2.94	4.50	5.68
7		0.92	0.99	1.94	2.71	4.11	3.94	5.67	5.11
8		0.72	1.02	2.11	2.50	2.78	2.39	3.91	5.36
	Mean	0.80	0.92	1.95	3.04	3.13	2.83	4.03	4.09
	SD	0.24	0.27	0.55	0.87	0.79	0.92	0.95	1.21
NX3 Light-cure Clear									
1		0.22	1.17	1.70	2.50	3.29	3.53	3.28	2.56
2		0.78	0.88	1.64	1.81	2.57	2.22	2.07	3.09
3		0.30	1.62	2.30	2.89	4.12	3.73	4.65	4.01
4		0.61	1.40	1.85	3.03	4.79	4.43	5.43	5.41
5		0.45	1.12	1.56	3.71	5.59	3.37	2.86	2.39
6		0.36	1.20	2.30	3.19	5.04	3.04	3.57	2.58
7		0.65	1.82	3.04	6.18	6.20	4.97	3.94	3.92
8		0.30	1.14	1.98	4.16	5.30	3.68	3.46	6.26
	Mean	0.46	1.30	2.05	3.43	4.61	3.62	3.66	3.78
	SD	0.19	0.28	0.46	1.23	1.14	0.78	0.97	1.33

NX3 Light-cure									
Yellow									
	1	0.37	0.70	1.36	2.07	2.85	3.09	4.35	3.43
	2	0.22	0.64	1.75	1.86	2.58	2.91	3.38	3.85
	3	0.20	0.30	1.95	2.34	2.66	3.14	4.20	3.59
	4	0.81	1.82	2.22	1.81	2.07	2.48	4.80	4.25
	5	0.71	0.22	1.35	2.33	2.28	2.80	4.24	3.73
	6	0.14	0.32	1.75	2.04	1.96	2.28	4.48	2.60
	7	0.96	1.14	2.53	2.30	2.98	3.13	4.62	5.61
	8	0.58	1.64	3.41	2.67	2.77	2.30	3.83	2.78
	Mean	0.50	0.85	2.04	2.18	2.52	2.77	4.24	3.73
	SD	0.29	0.58	0.64	0.27	0.35	0.34	0.45	0.93
AllCem Veneer (ACV)									
E-Bleach M									
	1	0.40	1.14	1.71	1.63	4.67	3.85	2.89	2.75
	2	0.24	0.65	0.32	1.37	3.96	3.87	1.94	3.82
	3	0.33	1.05	0.92	1.66	3.92	4.50	2.28	2.35
	4	0.54	0.10	0.14	1.90	3.15	3.02	1.67	4.33
	5	1.62	1.51	2.13	2.37	3.99	3.29	3.93	3.58
	6	1.05	1.61	2.12	1.92	4.82	3.92	3.09	4.93
	7	0.24	0.91	0.77	1.92	3.37	4.37	2.18	3.30
	8	1.12	1.17	1.61	2.22	3.80	3.26	4.59	5.06
	Mean	0.69	1.02	1.21	1.88	3.96	3.76	2.82	3.76
	SD	0.48	0.45	0.73	0.30	0.53	0.50	0.95	0.91

AllCem Veneer (ACV)								
Trans								
1	0.37	2.26	1.02	1.58	3.45	4.58	4.64	4.12
2	0.45	1.49	0.92	0.94	2.02	2.06	2.66	1.62
3	1.01	1.33	2.21	0.70	3.17	2.26	3.25	2.64
4	0.46	1.16	1.32	2.58	2.60	3.69	2.51	1.94
5	0.42	1.28	1.61	1.40	3.29	4.85	3.59	2.85
6	1.00	0.90	1.09	1.17	3.03	2.76	2.55	1.86
7	0.33	1.81	3.62	4.66	4.41	6.27	4.14	4.80
8	0.51	1.36	1.71	4.13	4.62	4.88	3.66	2.98
Mean	0.57	1.45	1.69	2.15	3.33	3.92	3.38	2.85
SD	0.26	0.39	0.83	1.41	0.81	1.39	0.73	1.05
AllCem Veneer (ACV)								
A1								
1	0.54	0.83	0.71	1.64	2.59	2.18	3.02	3.61
2	0.52	3.24	2.12	2.30	2.75	3.31	2.95	4.02
3	0.36	0.92	1.08	0.95	2.61	2.40	3.00	2.17
4	0.71	1.52	1.50	2.94	3.56	3.51	4.26	3.62
5	0.40	0.89	1.45	1.24	3.60	2.78	2.74	2.11
7	1.76	1.76	2.93	4.18	5.59	3.42	2.39	2.99
8	0.24	1.04	1.91	1.95	3.72	3.20	2.98	2.57
Mean	0.65	1.46	1.67	2.17	3.49	2.97	3.05	3.01
SD	0.47	0.80	0.67	1.02	0.97	0.49	0.54	0.70

NX3 Dual-cure White									
1		0.59	1.36	1.30	2.02	2.39	3.49	4.75	3.11
2		0.30	0.83	1.32	2.38	3.07	3.92	3.50	3.14
3		0.42	0.93	1.36	2.32	2.76	3.80	4.11	3.83
4		0.46	1.35	1.30	2.77	2.20	3.10	4.98	4.30
5		0.70	2.67	1.86	2.09	2.24	2.59	3.48	4.35
6		0.45	1.56	1.77	5.14	2.75	3.78	5.03	3.18
7		0.71	1.98	1.62	4.00	2.83	3.47	4.90	3.03
8		0.51	1.09	3.88	5.91	2.44	3.34	5.22	3.14
	Mean	0.52	1.47	1.80	3.33	2.58	3.44	4.50	3.51
	SD	0.13	0.57	0.81	1.41	0.29	0.41	0.66	0.53
NX3 Dual-cure Clear (claro)									
1		0.41	0.63	1.62	1.73	2.04	1.99	2.42	3.06
2		0.33	1.55	0.81	2.40	1.75	1.98	4.02	4.37
3		0.30	1.56	1.42	3.26	2.67	3.45	3.22	2.70
4		0.24	1.22	1.14	3.02	2.34	2.29	2.62	2.63
5		0.17	1.44	2.03	2.81	4.64	3.79	4.40	2.99
6		0.14	1.51	1.40	3.53	2.27	2.06	4.37	4.62
7		0.46	1.06	2.01	2.49	1.90	1.32	4.02	3.05
8		0.47	1.66	2.19	3.01	2.56	2.33	4.13	2.58
	Mean	0.32	1.33	1.58	2.78	2.52	2.40	3.65	3.25
	SD	0.12	0.32	0.45	0.53	0.85	0.76	0.74	0.74

NX3 Dual-cure									
Yellow									
1		0.14	1.71	0.90	2.56	1.53	1.58	4.43	2.81
2		0.20	1.69	0.95	2.49	2.19	1.85	4.74	2.93
3		0.65	2.57	0.81	1.73	1.78	1.80	2.16	2.58
4		0.44	1.55	2.16	2.21	1.28	1.53	4.88	3.99
5		0.10	1.55	1.42	2.44	1.56	2.25	4.46	5.14
6		0.81	2.37	1.53	2.56	2.24	2.91	3.37	5.17
7		0.28	2.15	1.62	2.35	2.60	1.61	2.74	4.88
8		0.37	1.51	0.67	2.28	3.48	2.59	4.37	4.52
	Mean	0.37	1.89	1.26	2.33	2.08	2.01	3.89	4.00
	SD	0.23	0.39	0.48	0.25	0.67	0.48	0.95	1.02
AllCem (AC)									
Trans									
1		0.41	1.90	1.75	2.08	2.26	4.73	3.07	2.81
2		0.37	1.77	2.57	2.32	2.31	5.66	3.22	2.97
3		0.87	1.97	2.44	3.87	3.61	5.80	4.45	3.66
4		0.64	2.04	2.41	6.22	4.50	6.12	5.02	4.76
5		0.47	2.11	2.31	5.11	4.96	5.87	5.61	6.54
6		0.51	2.53	2.51	5.66	3.64	5.16	5.03	4.89
7		0.33	2.80	2.79	5.89	4.07	6.07	5.74	5.32
8		0.86	3.69	3.12	6.71	5.06	5.36	6.73	5.16
	Mean	0.56	2.35	2.49	4.73	3.80	5.60	4.86	4.51
	SD	0.20	0.60	0.37	1.66	1.01	0.45	1.17	1.19

AllCem (AC)									
A1									
1		0.51	3.38	2.89	4.14	5.32	4.54	4.37	5.14
2		0.33	3.30	2.70	4.51	5.50	7.27	7.98	7.61
3		0.54	4.53	4.00	5.33	6.43	9.09	8.71	9.91
4		0.35	3.01	2.62	4.22	7.76	8.63	9.50	9.76
5		0.37	3.61	3.90	5.02	5.41	6.45	6.79	7.11
6		0.20	3.12	3.35	4.52	5.41	6.94	8.31	9.02
7		0.37	3.05	3.02	4.01	5.60	7.25	7.48	7.80
8		0.22	3.03	3.78	4.71	6.42	8.12	8.50	10.97
	Mean	0.36	3.38	3.28	4.56	5.98	7.29	7.70	8.42
	SD	0.11	0.48	0.52	0.42	0.79	1.33	1.47	1.75
AllCem (AC)									
A3									
1		0.58	2.24	1.80	2.69	7.44	8.49	7.34	7.28
2		0.61	3.00	2.85	4.44	3.62	5.03	4.66	3.86
3		1.10	2.95	2.06	4.24	5.22	5.52	5.96	6.23
4		0.71	2.58	2.20	3.66	5.84	5.94	6.23	6.40
5		0.30	2.03	1.70	3.23	4.32	5.14	4.22	4.47
6		1.08	2.26	1.56	2.89	6.03	6.20	7.13	7.42
7		1.12	1.27	2.01	3.30	6.72	6.62	4.38	5.21
8		0.41	2.55	2.17	3.72	2.55	3.69	4.31	4.71
	Mean	0.74	2.36	2.04	3.52	5.22	5.83	5.53	5.70
	SD	0.30	0.52	0.37	0.58	1.53	1.30	1.21	1.24
