# Supplementary material for: Color stability of ceramic veneers as a function of resin cement curing mode and shade: 3-year follow-up
Source: PLoS One. 2019 Jul 1;14(7):e0219183. doi: 10.1371/journal.pone.0219183 (PMC6602287; doi:10.1371/journal.pone.0219183)
Supplement: S2 File — (PDF) [file pone.0219183.s002.pdf]

Effect	Repeated Measures Analysis of Variance<br>Sigma-restricted parameterization<br>Effective hypothesis decomposition				
	SS	Degr. of Freedom	MS	F	p
Intercept	*2145,337*	*1*	*2145,337*	*978,5221*	*0,000000*
Light-cured resin cements	13,487	5	2,697	1,2303	0,312972
Error	87,697	40	2,192		
TIME	*420,143*	*6*	*70,024*	*148,8901*	*0,000000*
TIME*Light-cured resin cements	*56,222*	*30*	*1,874*	*3,9848*	*0,000000*
Error	112,873	240	0,470		

Cell No.	Tukey HSD test; variable DV_1<br>Homogenous Groups, alpha = ,05000<br>Error: Between MS = 2,1924, df = 40,000			
	Light-cured resin cements		DV_1<br>Mean	1
3		NX3-Yellow	2,373061	****
6		AC-A1	2,399592	****
4		AC-Trans	2,518750	****
5		AC-Bleach	2,555714	****
1		NX3-White	2,691429	****
2		NX3-Clear	2,979286	****

Cell No.	Tukey HSD test; variable DV_1<br>Homogenous Groups, alpha = ,05000<br>Error: Within MS = ,47031, df = 240,00					
	TIME	DV_1 Mean	1	2	3	4
1	DE1h	0,610870		****		
2	DE24h	1,147174			****	
3	DE30d	2,476522				****
5	DE1y	3,340870	****			
4	DE180d	3,521522	****			
6	DE2y	3,522826	****			
7	DE3y	3,545217	****			

Cell No.	Tukey HSD test; variable DV_1<br>Homogenous Groups, alpha = ,05000<br>Error: Within MS = ,47031, df = 240,00					
	TIME	DV_1<br>Mean	1	2	3	4
1	DE1h	0,610870		****		
2	DE24h	1,147174			****	
3	DE30d	2,476522				****
5	DE1y	3,340870	****			
4	DE180d	3,521522	****			
6	DE2y	3,522826	****			
7	DE3y	3,545217	****			

Cell No.	Tukey HSD test; variable DV_1<br>Homogenous Groups, alpha = ,05000 (Non-Exhaustive Search)<br>Error: Between; Within; Pooled MS = ,71632, df = 163,96														
	Light-cured resin cements	TIME	DV_1 Mean	1	2	3	4	5	6	7	8	9	10	11	12
8	NX3-Clear	DE1h	0,458750	****	****		****								
15	NX3-Yellow	DE1h	0,487143	****		****	****								
22	AC-Trans	DE1h	0,568750	****	****		****								
36	AC-A1	DE1h	0,647143	****	****	****									
29	AC-Bleach	DE1h	0,692500	****	****	****	****								
16	NX3-Yellow	DE24h	0,734286	****	****	****	****								
1	NX3-White	DE1h	0,800000	****	****	****	****	****							
2	NX3-White	DE24h	0,918750	****	****	****	****	****							
30	AC-Bleach	DE24h	1,017500	****	****	****	****	****							
9	NX3-Clear	DE24h	1,293750	****	****	****	****	****	****	****	****				
23	AC-Trans	DE24h	1,448750	****	****	****	****	****	****		****	****	****		
37	AC-A1	DE24h	1,457143	****	****	****	****	****	****	****			****	****	
31	AC-Bleach	DE30d	1,873750	****	****	****	****	****	****	****	****	****	****	****	****
17	NX3-Yellow	DE30d	2,107143		****			****	****	****	****	****	****	****	****
24	AC-Trans	DE30d	2,145000			****		****	****	****	****	****	****	****	****
38	AC-A1	DE30d	2,171429				****	****	****	****	****	****	****	****	****
18	NX3-Yellow	DE180d	2,482857					****	****	****	****	****	****	****	****
34	AC-Bleach	DE2y	2,821250						****	****	****	****	****	****	****
5	NX3-White	DE1y	2,831250						****	****	****	****	****	****	****
19	NX3-Yellow	DE1y	2,832857						****	****	****	****	****	****	****
28	AC-Trans	DE3y	2,851250							****				****	****
40	AC-A1	DE1y	2,971429								****	****			****
42	AC-A1	DE3y	3,012857									****			****
3	NX3-White	DE30d	3,043750									****	****	****	****
41	AC-A1	DE2y	3,048571									****			****
4	NX3-White	DE180d	3,127500											****	****
25	AC-Trans	DE180d	3,323750												****
27	AC-Trans	DE2y	3,375000												****
10	NX3-Clear	DE30d	3,433750												****
39	AC-A1	DE180d	3,488571												****
12	NX3-Clear	DE1y	3,621250												
13	NX3-Clear	DE2y	3,657500												
21	NX3-Yellow	DE3y	3,730000												
33	AC-Bleach	DE1y	3,760000												
35	AC-Bleach	DE3y	3,765000												
14	NX3-Clear	DE3y	3,777500												
26	AC-Trans	DE1y	3,918750												
32	AC-Bleach	DE180d	3,960000												
6	NX3-White	DE2y	4,027500												
7	NX3-White	DE3y	4,091250												
20	NX3-Yellow	DE2y	4,237143												
11	NX3-Clear	DE180d	4,612500												

Cell No.	Tukey HSD test; variable DV_1 [illegible]<br>Homogenous Groups, alpha = ,05000 (Non-Exhaustive Search)<br>Error: Between; Within; Pooled MS = ,71632, df = 163,96				
	13	14	15	16	17
8					
15					
22					
36					
29					
16					
1					
2					
30					
9					
23					
37					
31					
17	****				
24	****	****			
38	****	****			
18	****	****	****		
34	****	****	****	****	
5	****	****	****	****	
19	****	****	****	****	
28	****	****	****	****	
40	****	****	****	****	****
42	****	****	****	****	****
3	****	****	****	****	****
41	****	****	****	****	****
4	****	****	****	****	****
25	****	****	****	****	****
27	****	****	****	****	****
10	****	****	****	****	****
39	****	****	****	****	****
12	****	****	****	****	****
13	****	****	****	****	****
21		****	****	****	****
33	****	****	****	****	****
35	****	****	****	****	****
14	****	****	****	****	****
26			****	****	****
32			****	****	****
6			****	****	****
7			****	****	****
20				****	****
11					****
