# Supplementary material for: Color stability of ceramic veneers as a function of resin cement curing mode and shade: 3-year follow-up
Source: PLoS One. 2019 Jul 1;14(7):e0219183. doi: 10.1371/journal.pone.0219183 (PMC6602287; doi:10.1371/journal.pone.0219183)
Supplement: S3 File — (PDF) [file pone.0219183.s003.pdf]

Effect	Repeated Measures Analysis of Variance<br>Sigma-restricted parameterization<br>Effective hypothesis decomposition<br>SS	Degr. of Freedom	MS	F	p
Intercept	*4013,812*	*1*	*4013,812*	*1621,445*	*0,000000*
Dual-cured resin cements	*404,107*	*5*	*80,821*	*32,649*	*0,000000*
Error	103,969	42	2,475		
TIME	*776,341*	*6*	*129,390*	*215,491*	*0,000000*
TIME*Dual-cured resin cements	*173,187*	*30*	*5,773*	*9,614*	*0,000000*
Error	151,312	252	0,600		

Cell No.	Tukey HSD test; variable DV_1 [illegible]<br>Homogenous Groups, alpha = ,05000<br>Error: Between MS = 2,4755, df = 42,000				
	Dual-cured resin cements	DV_1 Mean	1	2	3
2	NX3-Clear	2,321071	****		
3	NX3-Yellow	2,368929	****		
1	NX3-White	2,763571	****		
4	AC-Trans	3,773036		****	
6	AC-A3	4,127500		****	
5	AC-A1	5,383571			****

Cell No.	Tukey HSD test; variable DV_1 [illegible]<br>Homogenous Groups, alpha = ,05000<br>Error: Within MS = ,60044, df = 252,00							
	TIME		DV_1<br>Mean	1	2	3	4	5
1	DI	E1h	0,477292			****		
2	DE	24h	2,129583				****	
3	DE	30d	3,541458	****				
4	DE1	30d	3,698125	****				
5	DI	E1y	4,427292					****
7	DI	E3y	4,898125		****			
6	DI	E2y	5,022083		****			

Cell No.	Tukey HSD test; variable DV_1 [illegible]<br>Homogenous Groups, alpha = ,05000 (Non-Exhaustive Search)<br>Error: Between; Within; Pooled MS = ,86830, df = 187,14														
	Dual-cured resin cements	TIME	DV_1 Mean	1	2	3	4	5	6	7	8	9	10	11	12
8	NX3-Clear	DE1h	0,315000	****	****										
29	AC-A1	DE1h	0,361250	****	****										
15	NX3-Yellow	DE1h	0,373750	****											
1	NX3-White	DE1h	0,517500	****	****	****									
22	AC-Trans	DE1h	0,557500	****	****	****		****							
36	AC-A3	DE1h	0,738750	****	****	****	****								
9	NX3-Clear	DE24h	1,328750	****	****	****	****	****	****						
2	NX3-White	DE24h	1,471250	****	****	****	****	****	****	****					
16	NX3-Yellow	DE24h	1,887500	****	****	****	****	****	****	****	****				
19	NX3-Yellow	DE1y	2,015000		****	****	****	****	****	****	****	****			
18	NX3-Yellow	DE180d	2,082500		****	****	****	****	****	****	****	****			
17	NX3-Yellow	DE30d	2,327500			****	****	****	****	****	****	****			
23	AC-Trans	DE24h	2,351250				****		****	****	****	****	****		
37	AC-A3	DE24h	2,360000					****	****	****	****	****	****		
12	NX3-Clear	DE1y	2,401250				****		****	****	****	****	****		
11	NX3-Clear	DE180d	2,521250				****		****	****	****	****	****		
4	NX3-White	DE180d	2,585000						****	****	****	****	****		
10	NX3-Clear	DE30d	2,781250						****	****	****	****	****	****	
14	NX3-Clear	DE3y	3,250000							****	****	****	****	****	****
3	NX3-White	DE30d	3,328750								****	****	****	****	****
30	AC-A1	DE24h	3,378750								****	****	****	****	****
5	NX3-White	DE1y	3,436250								****	****	****	****	****
7	NX3-White	DE3y	3,510000								****	****	****	****	****
38	AC-A3	DE30d	3,521250								****	****	****	****	****
13	NX3-Clear	DE2y	3,650000								****	****	****	****	****
25	AC-Trans	DE180d	3,801250									****	****	****	****
20	NX3-Yellow	DE2y	3,893750										****	****	****
21	NX3-Yellow	DE3y	4,002500										****	****	****
6	NX3-White	DE2y	4,496250											****	****
28	AC-Trans	DE3y	4,513750											****	****
31	AC-A1	DE30d	4,557500											****	****
24	AC-Trans	DE30d	4,732500												****
27	AC-Trans	DE2y	4,858750												****
39	AC-A3	DE180d	5,217500												
41	AC-A3	DE2y	5,528750												
26	AC-Trans	DE1y	5,596250												
42	AC-A3	DE3y	5,697500												
40	AC-A3	DE1y	5,828750												
32	AC-A1	DE180d	5,981250												
33	AC-A1	DE1y	7,286250												
34	AC-A1	DE2y	7,705000												
35	AC-A1	DE3y	8,415000												

Cell No.	Tukey HSD test; variable DV_1 [illegible]<br>Homogenous Groups, alpha = ,05000 (Non-Exhaustive Search)<br>Error: Between; Within; Pooled MS = ,86830, df = 187,14					
	13	14	15	16	17	18
8						
29						
15						
1						
22						
36						
9						
2						
16						
19						
18						
17						
23						
37						
12						
11						
4						
10						
14						
3						
30						
5	****					
7	****					
38						
13	****					
25	****	****				
20	****	****	****			
21	****	****	****			
6	****	****	****	****		
28	****	****	****	****		
31	****	****	****	****		
24	****	****	****	****		
27	****	****	****	****		
39	****	****	****	****		
41		****	****	****	****	
26			****	****	****	
42			****	****	****	
40				****	****	
32				****	****	
33					****	****
34						****
35						****
