# Supplementary material for: Color stability of ceramic veneers as a function of resin cement curing mode and shade: 3-year follow-up
Source: PLoS One. 2019 Jul 1;14(7):e0219183. doi: 10.1371/journal.pone.0219183 (PMC6602287; doi:10.1371/journal.pone.0219183)
Supplement: S4 File — (PDF) [file pone.0219183.s004.pdf]

CERTIFICATE OF ENGLISH EDITING

This document certifies that the paper listed below has been edited to ensure that the language is clear and free of errors. The edit was performed by professional editors at Editage, a division of Cactus Communications.The intent of the author's message was not altered in any way during the editing process. The quality of the edit has been guaranteed, with the assumption that our suggested changes have been accepted and have not been further altered without the knowledge of our editors.

Title Of The Paper

Color stability of ceramic veneers as a function of resin-cement curing mode and shade: 3-year follow-up

Authors

Janes Francio Pissaia Brunna Katyuscia de Almeida Guanaes Cibele Cândida de Almeida Kintopp Gisele Maria Correr Leonardo Fernandes da Cunha Carla Castiglia Gonzaga

Job Code

JAFRA_5

Signature

Vikas Narang

Vikas Narang,
Senior Vice President,
Operations-Author Services, Editage

Date of Issue
June 07, 2019

Editage, a brand of Cactus Communications, offers professional English language editing and publication support services to authors engaged in over 500 areas of research. Through its community of experienced editors, which includes doctors, engineers, published scientists, and researchers with peer review experience, Editage has successfully helped authors get published in internationally reputed journals. Authors who work with Editage are guaranteed excellent language quality and timely delivery.

INFORMATION SECURITY MANAGEMENT SYSTEM DNV·GL ISO/IEC 27001

CACTUS.

Contact Editage

Worldwide	Japan	Korea	China	Brazil	Taiwan
request@editage.com	submissions@editage.com	submit-korea@editage.com	fabiao@editage.cn	contato@editage.com	submitjobs@editage.com
+1 877-334-8243	+81 03-6868-3348	1544-9241	400-005-6055	0800-892-20-97	02 2657 0306
www.editage.com	www.editage.jp	www.editage.co.kr	www.editage.cn	www.editage.com.br	www.editage.com.tw
